# Stearoyl-CoA desaturase 1 inhibition induces ER stress-mediated apoptosis in ovarian cancer cells

**DOI:** 10.1186/s13048-024-01389-1

**Published:** 2024-04-02

**Authors:** Juwon Lee, Suin Jang, Jihye Im, Youngjin Han, Soochi Kim, HyunA Jo, Wenyu Wang, Untack Cho, Se Ik Kim, Aeran Seol, Boyun Kim, Yong Sang Song

**Affiliations:** 1 WCU Biomodulation, Department of Agricultural Biotechnology, Seoul National University, Seoul, Republic of Korea; 2 Cancer Research Institute, College of Medicine, Seoul National University, Seoul, Republic of Korea; 3 Department of Neurology and Neurological Sciences, Stanford University School of Medicine, Stanford, CA 94305 USA; 4 Paul F. Glenn Laboratories for the Biology of Aging, Stanford University School of Medicine, Stanford, CA 94305 USA; 5 Department of Medical Oncology, The First Affiliated Hospital, College of Medicine, Zhejiang University, Hangzhou, China; 6 Department of Obstetrics and Gynecology, College of Medicine, Seoul National University, Seoul, Republic of Korea; 7 Department of Obstetrics and Gynecology, Korea University College of Medicine, Seoul, Republic of Korea; 8 Department of SmartBio, College of Life and Health Science, Kyungsung University, Busan, Republic of Korea; 9 Present Address: Department of Obstetrics and Gynecology, Myongji Hospital, Hanyang University College of Medicine, Goyang, Republic of Korea

**Keywords:** Ovarian cancer, Lipid metabolism, SCD1, ER stress, Apoptosis

## Abstract

Ovarian cancer is a leading cause of death among gynecologic tumors, often detected at advanced stages. Metabolic reprogramming and increased lipid biosynthesis are key factors driving cancer cell growth. Stearoyl-CoA desaturase 1 (SCD1) is a crucial enzyme involved in de novo lipid synthesis, producing mono-unsaturated fatty acids (MUFAs). Here, we aimed to investigate the expression and significance of SCD1 in epithelial ovarian cancer (EOC). Comparative analysis of normal ovarian surface epithelial (NOSE) tissues and cell lines revealed elevated SCD1 expression in EOC tissues and cells. Inhibition of SCD1 significantly reduced the proliferation of EOC cells and patient-derived organoids and induced apoptotic cell death. Interestingly, SCD1 inhibition did not affect the viability of non-cancer cells, indicating selective cytotoxicity against EOC cells. SCD1 inhibition on EOC cells induced endoplasmic reticulum (ER) stress by activating the unfolded protein response (UPR) sensors and resulted in apoptosis. The addition of exogenous oleic acid, a product of SCD1, rescued EOC cells from ER stress-mediated apoptosis induced by SCD1 inhibition, underscoring the importance of lipid desaturation for cancer cell survival. Taken together, our findings suggest that the inhibition of SCD1 is a promising biomarker as well as a novel therapeutic target for ovarian cancer by regulating ER stress and inducing cancer cell apoptosis.

**Supplementary Information:**

The online version contains supplementary material available at 10.1186/s13048-024-01389-1.

## Introduction

Ovarian cancer is the deadliest gynecological cancer and the fifth leading cause of cancer-related female death in the United States [1]. Epithelial ovarian cancer (EOC) is the most common type of ovarian cancer, accounting for ∼ 90% of ovarian malignancies [2]. However, most cases of EOC are detected at the advanced stages, making early detection and diagnosis challenging. The absence of noticeable symptoms in the early stages further contributes to its elusive nature resulting in a high mortality rate of ovarian cancer [3–5]. Accumulating studies have indicated that the gaining of proliferative and metastatic phenotype was closely associated with alterations in a number of metabolic pathways [6]. These processes require enhanced production of energy and cellular building blocks such as proteins, nucleic acids, and lipids [7]. The Warburg effect, or aerobic glycolysis, is the most well-known metabolic phenotype in cancer cells, characterized by excessive glucose uptake and enhanced lactate generation under aerobic circumstances [8]. The malignant progression of ovarian cancer also brings a series of changes in its metabolism, including the metabolism of glucose, amino acids, and lipids, which is in favor of strengthening the malignancy of the disease [9, 10].

Fatty acids play an essential role in the development, proliferation, and survival of cancer cells [11, 12]. Proliferating cells, especially cancer cells, require a greater amount of metabolites for cell growth, and the abundance of lipids composing the plasma membrane is particularly essential for cell proliferation [13]. Regarding membrane biosynthesis, fatty acids are used as building blocks, including phospholipids and other lipids such as sterols and sphingolipids. Fatty acids are stored as triacylglycerides and act as secondary messengers in signal transduction. Interestingly, normal cells primarily use fatty acids from external sources, but cancer cells rely heavily on de novo synthesis for up to 95% of their fatty acid requirements, regardless of dietary lipid levels [14–16]. Lipogenic enzymes, including ATP citrate lyase, acetyl-CoA carboxylase, and fatty acid synthase (FASN), have elevated expression and activity in cancer cells, which exacerbates lipogenesis. Several previous investigations have demonstrated that inhibiting lipogenic pathways can inhibit the proliferation of cancer cells [9, 15, 16], suggesting that elevated lipogenesis is crucial for cancer cell survival.

Stearoyl-CoA desaturase 1 (SCD1), a rate-limiting enzyme, is a key regulator of *de novo* fatty acid synthesis. SCD1 converts saturated fatty acids (SFAs) into mono-unsaturated fatty acids (MUFAs), primarily palmitic acid (C16:0) to palmitoleic acid (C16:1) and stearic acid (C18:0) to oleic acid (C18:1), respectively [17]. SCD1 is abundantly expressed in a range of human malignancies, including breast [18], lung [19], liver [20], and ovarian cancers [21], relative to the corresponding normal tissues [22]. Elevated SCD1 levels are found to be associated with poor prognosis in liver and lung cancer patients [19, 20]. Furthermore, inhibiting SCD1 activity or expression in cancer cells reduces cell proliferation and promotes apoptosis [21, 23]. However, the functional relevance of SCD1 in ovarian cancer, as well as the molecular mechanism behind cancer cell death triggered by SCD1 inhibition, have not yet been fully elucidated.

In this study, we found that ovarian cancer cells exhibit high levels of SCD1 mRNA and protein expression relative to normal ovarian epithelial cells. Furthermore, genetic and pharmacological inhibition of SCD1 reduced cell proliferation and induced ER stress-mediated apoptosis in ovarian cancer cells without cytotoxic effects on normal ovarian epithelial cells and peripheral blood mononuclear cells (PBMCs). Finally, with the addition of exogenous oleic acid, the main product of SCD1 activity restored the suppression of cancer cell proliferation and the induction of ER stress-mediated apoptosis triggered by SCD1 inhibition. Altogether, our results strongly suggest that SCD1 could serve as an important biomarker as well as a therapeutic target for ovarian cancer.

## Materials and methods

### Cell culture

Human ovarian cancer cell lines PA-1, OVCAR-3, TOV112D, and SKOV-3 were purchased from the American Type Culture Collection (Rockville, MD), and SNU840 was obtained from the Korean Cell Line Bank (Seoul, Korea). A2780 was kindly gifted by Prof. Benjamin K. Tsang (University of Ottawa, Canada). Induced ovarian surface epithelial cell line IOSE385 was generously gifted from Prof. Young Kee Shin (Seoul National University, Korea) and SNU3297 and SNU3236 were provided by Prof. Ja-Lok Ku (Seoul National University, Korea), respectively. PA-1 was cultured in MEM (WelGENE, Seoul, Korea), and other cancer cell lines were cultured in RPMI1640 (WelGENE). TOV112D and normal ovarian cell lines were grown in DMEM/F12 (Gibco-BRL, Gaithersburg, MD). All media were supplemented with 10% fetal bovine serum (WelGENE), 100 Units/ml penicillin, and 100 µg/ml streptomycin (Gibco-BRL). All cells were cultivated at 37 °C in humidified conditions with 5% CO_2_. For assays, all cell lines were treated with CAY10566 or SCD1 siRNA for 24–48 h in a 1% serum-containing medium.

### Reagents and antibodies

CAY10566 was purchased from Cayman Chemical (Ann Arbor, MI). Oleic acid-BSA conjugate and fatty acid-free BSA were purchased from Sigma-Aldrich (St. Louis, MO). Antibodies used for western blotting were as follows: P-PERK (Thr981), PARP, and cleaved caspase-3 from Santa Cruz Biotechnology (Santa Cruz, CA), IRE1a and ATF4 from Cell Signaling Technology (Danvers, MA), SCD1 from Abcam (Cambridge, UK), CHOP (GADD153) from Thermo Fisher Scientific (Waltham, MA), and GAPDH from Ab Frontier (Seoul, Korea).

### Quantitative real-time PCR (qRT-PCR)

Total RNA was extracted using RNAiso Plus (TaKaRa, Tokyo, Japan), and the concentration of RNA was determined by Nano Drop2000 (Thermo Fisher Scientific). Complementary DNAs were synthesized from 1 µg of total RNA with oligo-dT primers and PrimeScript Reverse Transcriptase (TaKaRa). PCR was performed using QuantiSpeed SYBR No-ROX Kit (PhileKorea, Seoul, Korea) and the following specific primers: SCD1 sense 5’-CGA CGT GGC TTT TTC TTC TC-3’, antisense 5’-GGG GGC TAA TGT TCT TGT CA-3’ and GAPDH sense 5’-GAG TCA ACG GAT TTG GTC GT-3’, antisense 5’-TTG ATT TTG GAG GGA TCT CG-3’. The amplification conditions were as follows: an initial denaturation step at 95 °C for 3 min, followed by 45 cycles of denaturation at 94 °C for 5 s, annealing at 60 °C for 15 s, extension at 72 °C for 10 s, and a final extension step at 72 °C for 10 min. The relative gene expression levels were calculated using the comparative Ct method, and GAPDH was used as a reference gene.

### Cell viability assay

Cell viability was examined by MTT assay. For CAY10566 treatment, cells were seeded into 96-well plates at a density of 4,000–10,000 cells per well. The cells were treated with various concentrations of CAY10566 or DMSO (solvent control) for 24–48 h. For siRNA transfection, cells were seeded into 6-well plates to be 60–80% confluent at transfection. After 24 h, the cells were transfected with SCD1 siRNA or scrambled siRNA (negative control), incubated overnight, and then cultured into 96-well plates for 24–48 h. At the end of the treatment, cells were incubated with 50 µl of MTT solution (2 mg/ml) for 3 h at 37 °C in humidified conditions with 5% CO_2_ and subsequently solubilized in 100 µl of DMSO for 30 min. The optical density was measured at 540 nm using a Multiskan Ascent plate reader (Thermo LabSystems, Helsinki, Finland).

### Gas chromatography

The desaturase activity of SCD1 was measured by gas chromatography. CAY10566-treated cells were harvested by centrifugation and freeze-dried using a freeze dryer (LABCONCO, Kansas City, MO). The extraction and methylation of fatty acids were conducted as previously described [17]. For GC analysis, fatty acids were converted to their methyl esters (FAMEs). Gas chromatography was performed using an Agilent 7890 A GC system (Agilent Technologies, Wilmington, DE) equipped with a DB-23 capillary column (60 mm x 0.25 mm x 0.25 μm; Agilent Technologies). The GC conditions were as follows: the initial temperature was 50 °C for 1 min, then raised to 130 °C at 15 °C/min, to 170 °C at 8 °C/min, to 215 °C at 2 °C/min, and held for 10 min. The injector temperature was set at 250 °C, and the detector temperature was set at 280 °C. Pentadecanoic acid (C15:0) was used as an internal standard for quantification. The ratios of palmitoleic acid (C16:1) to palmitic acid (C16:0) and oleic acid (C18:1n9c) to stearic acid (C18:0) were determined for this study.

### Small-interfering RNA (siRNA) transfection

The SCD1 siRNA target sequence was 5’-GAGAUAAGU UGG AGA CGA UUU-3’. Scrambled siRNA was used as a negative control (Genolution, Seoul, Korea). Cells were transfected with the siRNA oligonucleotides (100 nM) using Lipofectamine RNAiMAX (Invitrogen Life Technologies, Carlsbad, CA) according to the manufacturer’s protocols. At 24 h post-transfection, the cells were used for cell proliferation assay, flow cytometry analysis, and western blot analysis.

### Isolation of peripheral blood mononuclear cells (PBMCs)

To determine the cytotoxic effect of CAY10566 on normal cells, buffy coats from healthy donors were collected under the approval of Seoul National University Hospital Institutional Review Board (C-1307-008-502). Human peripheral blood mononuclear cells were isolated from buffy coats by density-gradient centrifugation using Ficoll-Paque PLUS (GE Healthcare Life Sciences, Marlborough, MA) according to the manufacturer’s instructions. In brief, buffy coats were diluted in PBS, carefully layered on Ficoll-Paque PLUS, and centrifuged at 400 x g for 30 min at 20 °C. The PBMC layer was transferred to a clean centrifuge tube, washed twice with PBS, centrifuged at 200 x g for 15 min at 20 °C, and suspended in RPMI1640 supplemented with 10% FBS, 100 Units/ml penicillin, and 100 µg/ml streptomycin.

### Flow cytometry analysis

To analyze both floating and adherent cells, culture media containing floating cells were collected into a round-bottom tube (BD Falcon, San Jose, CA), and adherent cells were trypsinized, washed with cold PBS, and collected by centrifugation at 4 °C. All cells were stained with Annexin V-FITC and PI using Annexin V-FITC Apoptosis Detection Kit I (BD Biosciences, San Jose, CA) according to the manufacturer’s instructions. The stained cells were analyzed using a BD FACS Canto II flow cytometer (BD Biosciences) with BD FACS Diva software (BD Biosciences).

### Western blot analysis

Cells were lysed with the lysis buffer containing the premade 2X lysis buffer (150 mM NaCl, 10 mM Tris-HCI (pH 7.4), 1 mM EDTA, and 1 mM EGTA), 1% Triton X-100, 1 mM PMSF, 0.1% DCA, and 1X EDTA-free protease inhibitor cocktail (Roche Diagnostics, Indianapolis, IN). The concentration of protein was determined using BCA Protein Assay Kit (Thermo Fisher Scientific). 10–20 µg of proteins were loaded onto 6–15% SDS-PAGE gels for separation and transferred to nitrocellulose membranes (GE Healthcare Life Sciences) for detection by immunoblotting. The membranes were blocked with 5% skim milk in Tris-buffered saline containing 0.1% Tween-20, incubated with primary antibodies overnight at 4 °C, followed by incubation with HRP-conjugated secondary antibodies for 2 h at room temperature. Signals were visualized with the enhanced chemiluminescence detection kits, WESTSAVE up (AbFrontier) and ECL Select Western Blotting Detection Reagent (GE Healthcare Life Sciences).

### Organoid establishment

This study was approved by the Institutional Review Board of Seoul National University Hospital (IRB No. 2108-237-1251). Ovarian cancer tissue and ascites samples were collected with the consent of patients. Organoids were established with ovarian cancer acites as our previous study [24, 25]. The cells isolated from ascites were embedded in the in phenol red-free Matrigel Growth Factor Reduced Basement Membrane Matrix (BD Bioscience, CA, USA) and cultured for approximately 28 days. The culture conditions included consistent media changes every 2–3 days using Advanced DMEM/F12 (Gibco, MD, USA) supplemented with HEPES (10mM; Gibco, Gaithersburg, MD, USA), 1× GlutaMax (Gibco, Gaithersburg, MD, USA), 1× N2 (Invitrogen, CA, USA), 1× B27 (Invitrogen, CA, USA), β-Estradiol (1 mM; Sigma-Aldrich, St. Louis, USA), nicotinamide (1 mM; Sigma-Aldrich, St. Louis, USA), recombinant human Noggin (10 ng/mL; PeproTech, Rocky Hill, NJ, USA), recombinant R-Spondin1 (10 ng/mL; PeproTech, Rocky Hill, NJ, USA), EGF (10 ng/mL; Invitrogen, CA, USA), FGF2 (10 ng/mL; PeproTech, Rocky Hill, NJ, USA), FGF10 (10 ng/mL; PeproTech, Rocky Hill, NJ, USA), Y-27,632 dihydrochloride (10 µM; Sigma-Aldrich, St. Louis, USA), SB431542 (0.5 µM; Sigma-Aldrich, St. Louis, USA), and N-acetylcysteine (1mM; Sigma-Aldrich, St. Louis, USA). Bright field images of organoids were taken using EVOS M5000 (ThermoFisher Scientific).

### Organoid viability assay

For organoid cell viability assay, organoids MTT assay was carried out as our previous study [24]. Briefly, fully grown organoids embedded in Matrigel were seeded in 24-well plates and each well was treated with 500 µl of MTT reagent initially. Following a 3-hour incubation, 200 µl of DMSO was added, and the Matrigel was dissociated by pipetting. The plates were placed on an orbital shaker and incubated for 30 min at room temperature. Subsequently, the optical density values were measured at a wavelength of 540 nm with a Multi-Scan Spectrum (Thermo Scientific, NH, USA).

### Tissue microarray (TMA) construction and immunohistochemistry (IHC) staining

At Seoul National University Hospital, specimens of patients who have agreed to donate human materials are stored in the pathology department after surgery. We were provided with tissue microarray (TMA) blocks with personal information removed through hospital regulations. (IRB: 1807-037-956). All of the cases had available formalin-fixed paraffin-embedded (FFPE) specimens of primary and metastatic tumors and adjacent normal tissue which were obtained from biopsy. TMAs of ovarian cancer specimens were constructed using two cores (2 mm in diameter) from each specimen embedded in recipient paraffin blocks which include multiple sections of normal and cancer tissues of 72 ovarian cancer patients in Seoul National University Hospital using a trephine device (Superbiochips Laboratories, Seoul, Republic of Korea). TMAs were sectioned at a thickness of 4 μm and stained according to the manufacturer’s recommendations using the Benchmark XT Staining Systems (Ventana, Tucson, AZ). IHC staining was conducted using a rabbit polyclonal antibody against SCD1 (1:200, bs-3787R, Bioss, Woburn, MA). The intensity of positively stained cells was scored as follows: 0 (negative), 1 (weekly positive), 2 (moderately positive), 3 (strongly positive).

### Statistical analysis

All data were expressed as mean ± SEM of three independent experiments. The statistical significance of differences was determined using student’s t-test for two groups and two-way ANOVA with Šídák’s multiple comparisons test for over three groups. All statistical analyses were performed using IBM SPSS Statistics 22 software (SPSS Inc., Chicago, IL) and GraphPad Prism 9 (GraphPad Software, La Jolla, CA). For all analyses, differences with *p*-values < 0.05 were considered statistically significant.

## Results

### Patient derived-EOCs show higher expression levels of SCD1 than normal ovarian tissues

**Fig. 1:**
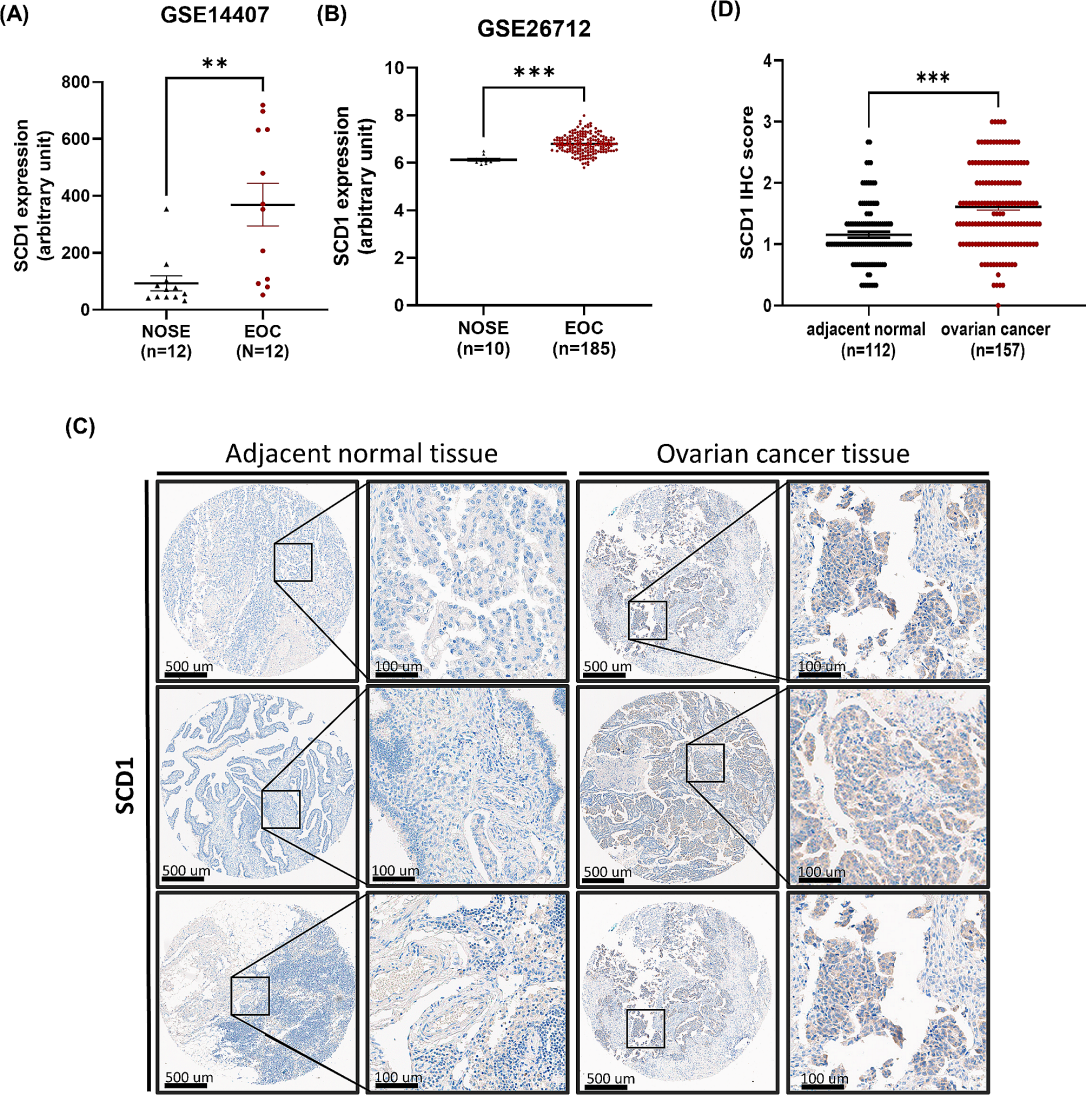
SCD1 is highly expressed in ovarian cancer tissues compared to normal tissues. (**A, B**) The microarray data for *SCD1* mRNA expression in normal ovarian surface epithelial (NOSE) and epithelial ovarian cancer (EOC) tissue samples was obtained from Gene Expression Omnibus (GEO) database, including (**A**) GSE14407 and (**B**) GSE26712. (**C**) Representative IHC images showing SCD1 expression pattern in adjacent normal tissues and ovarian cancer tissues in TMA sections. SCD1 expression was scored as 0, 1+, 2+, or 3 + based on staining intensity. (**D**) Different IHC score of SCD1 protein expression between adjacent normal and ovarian cancer tissues. Values are presented as means ± SEM (**p < 0.01; ***p < 0.001)

SCD1 is increased in many carcinomas compared to their normal tissues, and overexpression of SCD1 in cancer cells has been shown to promote cell proliferation and inhibit apoptosis [26]. Therefore, we screened the *SCD1* expression using the microarray data from the NCBI GEO database (accession number GSE 14,407 (Fig. 1A) and GSE 26,712 (Fig. 1B)) and evaluated the expression levels of *SCD1* in epithelial ovarian cancer (EOC) cells and normal ovarian surface epithelial (NOSE) cells. Both microarray data showed that *SCD1* gene expression was highly upregulated in EOC cells compared to NOSE cells (Fig. 1A and B). Next, we carried out IHC analysis of the 157 tumors and 112 normal tissues comprising samples of 72 ovarian cancer patients to validate transcriptomic findings at the protein level. The representative results elucidated that SCD1 was significantly elevated in tumor tissues compared to normal tissues (Fig. 1C and D).

**Fig. 2:**
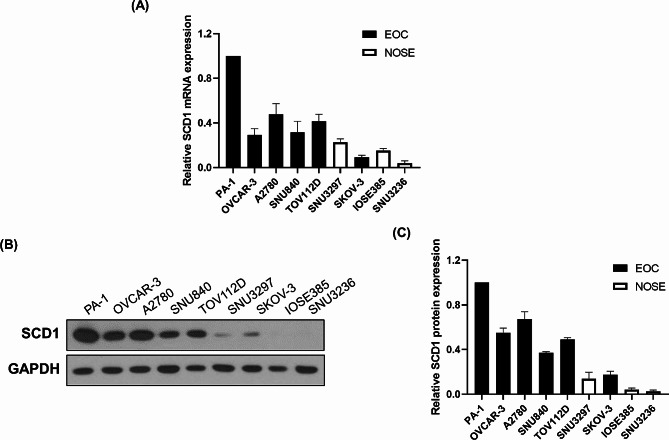
SCD1 expression is significantly elevated in EOC cell lines compared to NOSE cell lines. (**A**) The mRNA expression levels of SCD1 in EOC cell lines and NOSE cell lines. SCD1 mRNA levels were analyzed by qRT-PCR analysis and normalized to GAPDH mRNA levels. (**B**) The protein expression levels of SCD1 in EOC cell lines and NOSE cell lines were detected by western blot analysis. (**C**) SCD1 protein levels were quantified by densitometry using ImageJ software followed by normalization to GAPDH protein levels. Data are presented as the mean ± SEM of three independent experiments

We further investigated whether SCD1 expression is universally elevated in ovarian cancer cell lines. The qRT-PCR analysis revealed that the expression level of *SCD1* mRNA in EOC cells was much higher than in NOSE cells (Fig. 2A). Consistent with the mRNA expression level, western blot analysis confirmed the overexpression of SCD1 protein only in EOC cells (Fig. 2B and C). These results demonstrate that SCD1 is highly expressed at both mRNA and protein levels in EOC cells but not in NOSE cells. Based on these results, we chose two cancer cell lines displaying the highest expression of SCD1 (PA-1 cells), and the lowest expression of SCD1 (SKOV-3 cells), for the subsequent experiments.

### Genetic and pharmacologic inhibition of SCD1 selectively decreases ovarian cancer cell viability

**Fig. 3:**
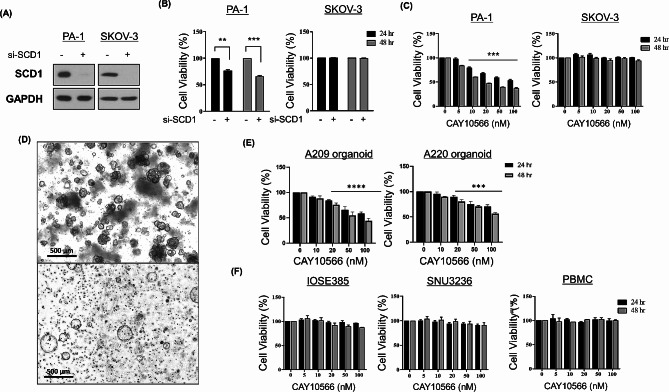
SCD1 inhibition reduces cancer cell proliferation without cytotoxic effect on normal cells. (**A**) PA-1 and SKOV-3 cells were transfected with SCD1 siRNA (100 nM) or scrambled siRNA (100 nM) as a negative control. After 72 h, the ablation of SCD1 protein was determined by western blot analysis. GAPDH was used as a loading control. (**B**) PA-1 and SKOV-3 cells were transfected with SCD1 siRNA (100 nM). At 72 h post-transfection, cell viability was analyzed using MTT assay. (**C**) PA-1 and SKOV-3 cells were treated with various concentrations (0, 5, 10, 20, 50, and 100 nM) of CAY10566 or DMSO (solvent control) for 24–48 h. Cell viability was measured by MTT assay. (**D**) Bright field microscopy images of patient-derived ovarian cancer organoids A209 (top) and A220 (bottom) after 28 days of culture. Scale bar = 500 μm. (**E**) Ovarian cancer organoids A209 and A220 were treated with varying concentrations (0, 10, 20, 50, and 100 nM) of CAY10566 or DMSO (solvent control) for 24–48 h. Cell viability was assessed using the organoid MTT assay. (**F**) CAY10566 were treated in NOSE cell lines (IOSE385 and SNU3236) and peripheral blood mononuclear cells (PBMCs) with different concentrations of CAY10566. After 24–48 h, cell viability was examined by MTT assay. All data were described as mean ± SEM of three independent experiments (**p < 0.01; ***p < 0.001; ****p < 0.0001)

To elucidate the biological function of SCD1 in ovarian cancer, we introduced short interfering RNA (siRNA) against *SCD1* into PA-1 and SKOV-3 cells by transfection. Genetic knockdown of *SCD1* using siRNA fully inhibited SCD1 expression in both ovarian cancer cells (Fig. 3A). The inhibitory effect of siRNA against *SCD1* on cell viability was observed in PA-1 cells but not in SKOV-3 cells (Fig. 3B). Likewise, the treatment of CAY10566, a small-molecule inhibitor of SCD1, significantly reduced cell viability in a concentration-dependent manner in PA-1 cells. In contrast, no appreciable effect of CAY10566 on cell viability was observed in SKOV-3 cells (Fig. 3C). In our earlier result, we found that SCD1 is overexpressed in ovarian cancer. To delve deeper into the effects of SCD1 inhibition, we conducted MTT on CAY10566 using patient-derived ovarian cancer organoids (Fig. 3D). Organoids derived from A209 and A220, ovarian cancer patients-derived ascites, were treated with CAY10566 for 24 and 48 h in a dose-dependent manner. CAY10566 treatment significantly reduced cell viability particularly from the concentration of more than 20 nM, in both organoids (Fig. 3E). These findings further underscore the potential therapeutic impact of SCD1 inhibition in ovarian cancer supporting the clinical relevance of our approach using patient-derived organoids. Moreover, to evaluate the effect of SCD1 inhibition on normal cell growth, we treated various concentrations of CAY10566 to NOSE cell lines (SNU3297, IOSE385 and SNU3236) and PBMC. CAY10566 failed to affect the proliferation of normal cells (Fig. 3F), suggesting that SCD1 inhibition probably has cancer-specific cytotoxicity.

### Inhibition of SCD1 induces ER stress-mediated apoptosis via SFA accumulation in EOC cells

**Fig. 4:**
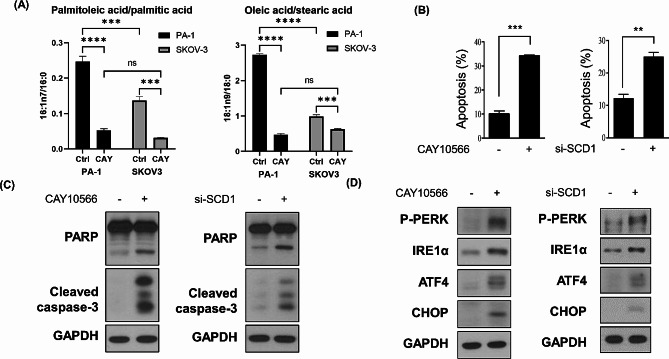
MUFA/SFA ratio alteration induced by SCD1 inhibition leads to ER stress-mediated apoptosis. (**A**) PA-1 and SKOV-3 cells were treated with the IC50 value of CAY10566 for PA-1 cells (20 nM) for 48 h. Desaturase activity of SCD1 was estimated as the ratios of palmitoleic acid (C16:1n7) to palmitic acid (C16:0) and oleic acid (C18:1n9c) to stearic acid (C18:0) using gas chromatography. The statistical analysis was conducted using a two-way ANOVA followed by Šídák’s multiple comparisons test. (**B**) PA-1 cells were transfected with SCD1 siRNA (100 nM) or treated with CAY10566 (20 nM) for 48 h. The percentage of apoptotic cells was determined by flow cytometry analysis using Annexin V-FITC and PI staining. (**C**) PA-1 cells were transfected with SCD1 siRNA (100 nM) or treated with CAY10566 (20 nM) for 48 h. The expression levels of apoptosis marker proteins, PARP and cleaved caspase-3, were detected by western blot analysis. GAPDH was used as a loading control. (**D**) PA-1 cells were transfected with SCD1 siRNA (100 nM) or treated with CAY10566 (20 nM) for 48 h. The expression levels of ER stress marker proteins, p-PERK, IRE1α, ATF4, and CHOP, were examined by western blot analysis. GAPDH was used as a loading control. All data were described as mean ± SEM of three independent experiments (**p < 0.01; ***p < 0.001; ****p < 0.0001)

Changes in the degree of fatty acid unsaturation in the cell membrane dramatically affect membrane fluidity and protein dynamics [7]. Multiple studies have shown that inhibition of SCD1 leads to SFA accumulation and depletion of MUFAs, which may induce ER stress and activation of UPRs, ultimately leading to cell death [27, 28]. Therefore, we first examined the change in fatty acid unsaturation due to SCD1 inhibition. CAY10566-mediated inhibition of SCD1 activity was confirmed by gas chromatography, followed by calculating the ratios of palmitoleic acid to palmitic acid (C16:1n7/C16:0) and oleic acid to stearic acid (C18:1n9c/C18:0). The accumulation of SFAs and the depletion of MUFAs by CAY10566 treatment were more pronounced in PA-1 cells comparerd to SKOV-3 cells (Fig. 4A).

Next, we examined whether SCD1 inhibition triggers apoptotic cell death in ovarian cancer cells by using Annexin V-FITC and PI staining flow cytometry analysis. Interestingly, CAY10566 treatment and SCD1 siRNA transfection dramatically induced apoptosis in PA-1 cells (Fig. 4B). Western blot analysis also revealed that the expression levels of apoptosis marker proteins, PARP and cleaved caspase-3, were elevated on CAY10566 treatment and siRNA transfection (Fig. 4C). To further investigate SCD1-induced apoptosis in ovarian cancer cells, we analyzed the expression levels of ER stress marker proteins, two master regulators (P-PERK, IRE1α) and their downstream effectors (ATF4 and CHOP). Western blot analysis showed that the blockade of SCD1 activity by CAY10566 strongly upregulated the ER stress-related proteins, suggesting the induction of ER stress and activation of the UPR (Fig. 4D). In addition, the genetic depletion of *SCD1* by siRNA triggered ER stress by increasing the expression levels of ER stress marker proteins (Fig. 4D). Using A2780, another SCD1 high-expressing cell line, we observed apoptosis and ER stress markers following CAY10566 treatment. Western blot analysis revealed that CAY10566 treatment increased apoptosis and ER stress marker proteins in A2780 cells (Figure S1). These findings demonstrate that ER stress can be induced by inhibiting desaturase activity of SCD1 or knockdown of *SCD1*.

### Supplementation with oleic acid reverses the proliferative defect and ER stress-mediated apoptosis triggered by SCD1 inhibition

**Fig. 5:**
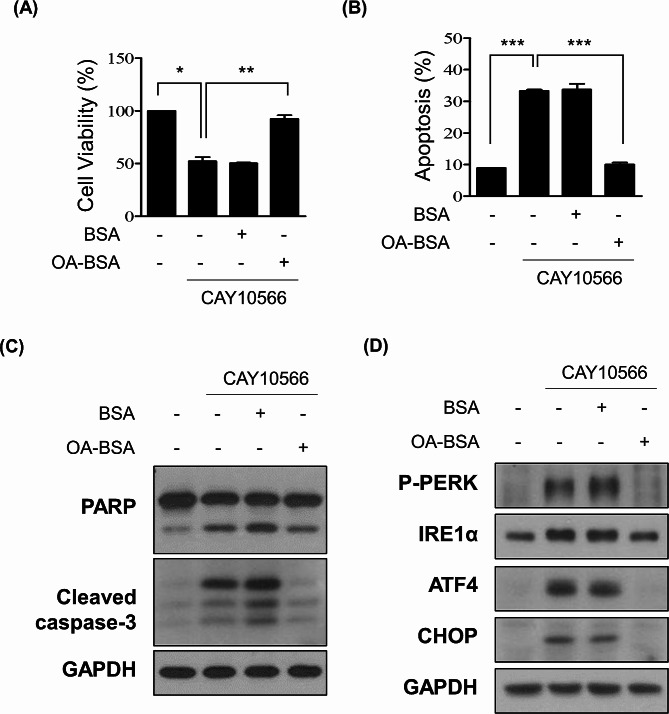
Addition of exogenous oleic acid rescued ER stress-mediated apoptosis triggered by SCD1 inhibition. (**A**) PA-1 cells were treated with CAY10566 (20 nM) with oleic acid-BSA (10 mM) or fatty acid-free BSA (10 µM) as vehicle control for 48 h. Cell viability was analyzed by MTT assay. Results were presented as the percentage of total cell number compared to DMSO-treated control. (**B**) PA-1 cells were treated with CAY10566 (20 nM) with or without oleic acid-BSA (10 µM) for 48 h. The percentage of apoptotic cells was calculated by flow cytometry analysis using Annexin V-FITC and PI staining. (**C**) Inhibition of CAY10566-mediated PARP and caspase-3 cleavage by supplementation with oleic acid. PA-1 cells were treated with CAY10566 (20 nM) with or without oleic acid-BSA (10 mM) for 48 h. The expression levels of cleaved PARP and caspase-3 were assessed by western blot analysis. GAPDH was used as a loading control. (**D**) PA-1 cells were treated with CAY10566 (20 nM) with or without oleic acid-BSA (10 µM) for 48 h. The expression levels of P-PERK, IRE1α, ATF4, and CHOP were determined by western blot analysis. GAPDH was used as a loading control. All data were described as mean ± SEM of three independent experiments (*p < 0.05; **p < 0.01; ***p < 0.001)

Subsequently, we further corroborated whether the ER-stress-mediated apoptosis induced by SCD1 inhibition was due to MUFA depletion. PA-1 cells were treated with CAY10566 with or without oleic acid conjugated to BSA (OA-BSA) for 48 h. The CAY10566-induced proliferative defect was completely rescued by oleic acid (Fig. 5A). Additionally, we assessed the effect of oleic acid on apoptotic cell death caused by SCD1 suppression. CAY10566-induced apoptosis was recovered by the addition of oleic acid (Fig. 5B). Western blot analysis also confirmed that the cleavage of PARP and caspase-3 was blocked by the addition of oleic acid (Fig. 5C). Furthermore, we determined whether exogenous oleic acid prevents ER stress triggered by CAY10566. As demonstrated in Fig. 4D, the addition of oleic acid resulted in a reduction of UPR proteins activated by treatment of CAY10566. (Fig. 5D). These findings suggest that the addition of exogenous oleic acid rescues the cells from ER stress-mediated apoptosis exerted by SCD1 inhibition. To further confirm the effect of oleic acid on ER stress and apoptosis in ovarian cancer, PA-1 cells were treated with oleic acid without SCD1 inhibition. Treatment with oleic acid solo had no effect on protein expression of ER stress and apoptosis markers (Figure S2).

## Discussion

Similar to previous studies, our results provide further evidence supporting the critical role of SCD1 in ovarian cancer survival. In this study, we confirmed that SCD1 is highly expressed in ovarian cancer cells and tissues compared to normal ones. We further examined the proliferative effect of SCD1 on cancer cells using CAY10566, an inhibitor of SCD1, and siRNA against *SCD1*. Consistent with the previous reports, inhibition of SCD1 decreased cancer cell proliferation [29, 30], but did not affect normal cell viability, suggesting cancer-specific cytotoxicity of SCD1 suppression. Lastly, we confirmed that inhibition of SCD1 caused ER-stress-mediated apoptosis by inhibiting MUFA accumulation.

Accumulating studies have highlighted the role of imbalanced saturation of fatty acid in increasing ER stress [28, 31–33]. The altered lipid environment in the ER membrane compromises the correct folding of newly synthesized proteins entering the ER [33]. Consequently, some of these proteins fail to attain their native and functional conformation and become misfolded. The presence of misfolded proteins in the ER lumen triggers the activation of the UPR signaling pathway. While the UPR initially aims to restore ER homeostasis, prolonged or severe ER stress leads to apoptosis, contributing cellular dysfunction and potentially impacting overall tissue or organ function [33]. SFAs, such as palmitic acid and stearic acid, have been shown to induce ER stress and apoptosis [28]. On the other hand, unsaturated fatty acids like oleic acid and linoleic acid exhibit a protective effect by maintaining ER membrane stability and reducing ER stress [33]. Several studies have confirmed that the downregulation of SCD1 causes UPR or ER stress in various diseases, such as cancers and obesity [34–36]. Changes in lipid saturation can disrupt lipid homeostasis and fluidity, leading to the accumulation of unfolded or misfolded proteins in the ER, thereby triggering the unfolded protein response (UPR) pathway [37, 38]. Understanding the impact of lipid saturation on ER stress induction is crucial for elucidating the molecular mechanisms underlying various diseases associated with lipid metabolism and ER stress-related pathologies. In our study, in cells with elevated SCD1 expression, there was a higher ratio of MUFA/SFA compared to cells with lower SCD1 expression. When inhibiting SCD1 with the same dose of CAY10566, cells with initially low SCD1 showed no distinctive changes in cell viability, while cells with high SCD1 exhibited a significant reduction in proliferation. This observation suggests that the impact of SCD1 inhibition on cell viability is more pronounced in cells with higher SCD1 expression, where the alteration in the balance between MUFA and SFA appears to play a crucial role. The higher MUFA/SFA ratio in cells with high SCD1 may indicate a greater reliance on SCD1-mediated MUFA synthesis for cell proliferation. By inhibiting SCD1 in ovarian cancer cells with highly expressing SCD1, the disrupted lipid metabolism overloads the ER capacity and subsequently triggers apoptosis. This mechanism offers a potential therapeutic strategy for targeting ovarian cancer cells with high SCD1 expression.

Numerous studies have revealed that lipogenic enzymes, including SCD1, are upregulated in various cancers, including ovarian cancer, and inhibition of the enzymes causes cancer cell death [29, 30]. In addition, the interconnection between lipid metabolism and apoptosis is supported by evidence highlighting the importance of lipid and lipid metabolism enzymes [39]. . Lipids are crucial for the recruitment of effectors to membranes, and the autophagic apparatus is regulated by lipids or enzymes involved in lipid metabolism during vital phases [40]. Our findings that SCD1 was highly expressed in EOC cells and inhibition of SCD1 induces apoptosis are consistent with previous studies reporting elevated SCD1 expression in various cancer types and SCD1 inhibition may also be used as a potential therapeutic target. CAY10566 inhibited the proliferation and induced apoptosis in colorectal cancer cells [23]. In liver cancer cells, CAY10566 induced apoptosis by regulating autophagy [20]. A preclinical study on lung cancer also showed that CAY10566 suppressed metastasis and increased the overall survival of mice [41]. Likewise, CAY10566 inhibited the proliferation of ovarian cancer stem cell by diminishing the stemness and tumor formation in mice [42]. Similar to our study, SCD1 inhibition with CAY10566 in ovarian cancer cells induced ER stress, leading to increased cell apoptosis [43]. The study extensively validated the induction of ER stress by SCD1 and found out XBP1s induced via IRE1 as a key mediator in the ER stress response upon SCD1 inhibition. While this study closely parallels our findings, there are distinctive points of comparison. Notably, the ovarian cancer cell lines utilized in this study differ significantly from those employed in our research. Additionally, our study provides a robust confirmation of SCD1 overexpression in ovarian cancer tissues across 269 tissues through immunohistochemistry (IHC). Furthermore, we investigated the potential toxicity of SCD1 inhibition using CAY10566 on normal cells. Subsequently, both studies affirm that SCD1 inhibition induces ER stress leading to apoptosis in ovarian cancer. This collective reinforcement of the proposition strengthens the potential therapeutic viability of targeting SCD1 in ovarian cancer. Indeed, the anti-cancer effects of SCD1 inhibition have been demonstrated not only with CAY10566 but also with various other inhibitors across multiple cancer types. A939572, another orally available piperidine-aryl urea-based small molecule inhibitor of SCD1, showed anticancer effects similar to those observed in CAY10566 treatment [44]. Accumulating studies have demonstrated that the inhibition of SCD1 by A939572 leads to the suppression of cancer cell proliferation and the induction of apoptosis in various types of solid cancers, including thyroid cancer [45], renal cell carcinoma [46], bladder cancer [47] and breast cancer [48]. Our study corroborates with these studies that SCD1 inhibition leads to reduced cell proliferation and increased apoptosis in cancer cells [26, 49–52]. Thus, the present findings further support the notion that SCD1 plays a critical role in promoting cancer cell survival and proliferation across different tumor types.

Previous studies in vitro and in vivo have shown promising results for SCD1 inhibitors as a therapeutic strategy for cancer and metabolic diseases. In current preclinical studies, 21 commercially available SCD1 inhibitors have been tested in cancer and metabolic diseases such as type 2 diabetes and hepatic steatosis, and nearly half of them have demonstrated inhibitory effects on cancer cell proliferation and tumor growth [49, 53]. A939572 has been broadly investigated in cancer research in both in vitro and in vivo models [49]. Other SCD1 inhibitors, including CAY10566, MF-438, and CVT-11,127, have been tested as anticancer agents and have shown suppressive effects on cancer cell proliferation [54]. In addition, targeted inhibition of SCD1 has been shown to be effective in preventing diet-induced obesity, hepatic steatosis, and other metabolic disorders [55, 56]. However, the use of SCD1 inhibitors may lead to mechanism-based adverse events, such as eye dryness, hair loss, and skin dryness, due to the critical role of SCD1 in the production of sebum by the sebaceous glands [54]. Therefore, new SCD1 inhibitors that are administrable as “pro-drugs” have been developed to overcome these adverse events [54]. Overall, SCD1 inhibitors show promise as a therapeutic strategy for cancer and metabolic diseases, but further research is needed to optimize their efficacy and minimize adverse effects. There are several clinical trials for SCD1 inhibitors. GSK1940029 gel, a novel SCD1 inhibitor, is being developed as a potential treatment for acne, and a phase 1 randomized, placebo-controlled trial has been conducted to assess its irritation potential [57]. In addition, Mayo Clinic Comprehensive Cancer Center Research has developed a novel small molecule inhibitor MTI-301 blocking the activity of SCD1, which promotes fatty acid synthesis in cancer cells (Project number: 4R44CA272064-02). Nonetheless, the current status of these clinical trials is not specified in the search results. Overall, while there are ongoing clinical trials for SCD1 inhibitors, more research is needed to determine their efficacy and safety in treating various diseases. In our study, we performed IHC on a total of 157 ovarian cancer tissues and 112 adjacent normal tissues obtained from 72 ovarian cancer patients. The patient cohort included 36 cases of high-grade serous ovarian cancer, 11 cases of clear cell carcinoma, 9 cases of mucinous carcinoma, 5 cases of endometrioid carcinoma, and 1 case of low-grade serous ovarian cancer. We observed an increased expression of SCD1 in ovarian cancer tissues compared to adjacent normal tissues. Notably, while clinical trials investigating SCD1 inhibitors have been propelled, no clinical trials have specifically targeted ovarian cancer. Therefore, our research findings suggest that SCD1 inhibition has the potential to be an ideal candidate for targeting ovarian cancer, indicating a potential therapeutic benefit in this specific cancer subtype.

In conclusion, our study corroborates previous research showing elevated SCD1 expression in cancer and validates the anti-cancer effects observed upon SCD1 inhibition. These findings underscore the potential of SCD1 as both a promising biomarker and a novel therapeutic target for ovarian cancer. Furthermore, our results with a large-scale validation of SCD1 protein expression in patient-derived ovarian cancer tissues emphasize the need for further investigations into SCD1 inhibitors as potential treatment options for ovarian cancer therapy. By shedding light on the role of SCD1 in cancer, our study contributes to the expanding knowledge surrounding lipid metabolism pathways and provides valuable insights for the development of targeted therapies in the field of ovarian cancer.

### Electronic supplementary material

Below is the link to the electronic supplementary material.

Supplementary Material 1

## Data Availability

No datasets were generated or analysed during the current study.

## Abbreviations

SCD1: Stearoyl-CoA desaturase 1
SFA: Saturated fatty acid
MUFA: Mono-unsaturated fatty acid
EOC: Epithelial ovarian cancer
NOSE: Normal ovarian surface epithelial
GEO: Gene expression omnibus
PBMC: Peripheral blood mononuclear cell
ER stress: Endoplasmic reticulum stress
UPR: Unfolded protein response
PARP: Poly ADP-ribose polymerase
PERK: Protein kinase RNA-like endoplasmic reticulum kinase
IRE1α: Inositol-requiring enzyme 1α
ATF4: Activating transcription factor 4
CHOP: C/EBP (CCAAT-enhancer-binding protein) homologous protein
GAPDH: Glyceraldehyde 3-phosphate dehydrogenase
OA-BSA: Oleic acid conjugated to bovine serum albumin
FASN: Fatty acid synthase
