# Supplementary material for: Stearoyl-CoA desaturase 1 inhibition induces ER stress-mediated apoptosis in ovarian cancer cells
Source: J Ovarian Res. 2024 Apr 2;17:73. doi: 10.1186/s13048-024-01389-1 (PMC10988872; doi:10.1186/s13048-024-01389-1)
Supplement: Supplementary file 1 — Supplementary Material 1 [file 13048_2024_1389_MOESM1_ESM.docx]

**Supplementary Figures:**


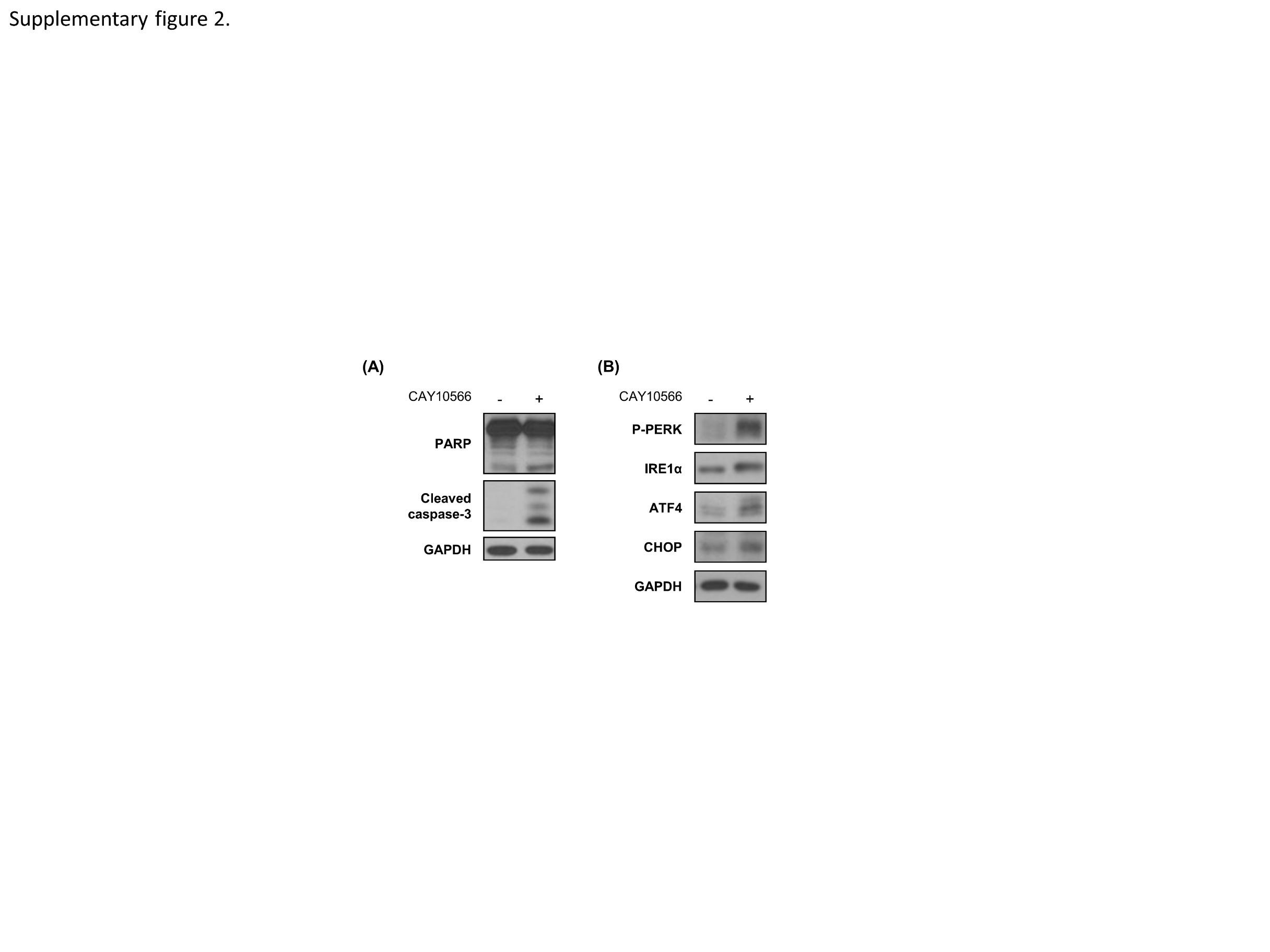


**Figure S1. CAY10566 increased apoptosis and ER stress in A2780 cells.** A2780 cells were treated with CAY10566 (20 nM) for 48 hours and protein expression of apoptosis and ER stress markers was examined by western blot analysis. (A) The expression levels of apoptosis marker proteins, PARP and cleaved caspase-3. (B) The expression levels of ER stress marker proteins, p-PERK, IRE1α, ATF4, and CHOP. GAPDH was used as a loading control.


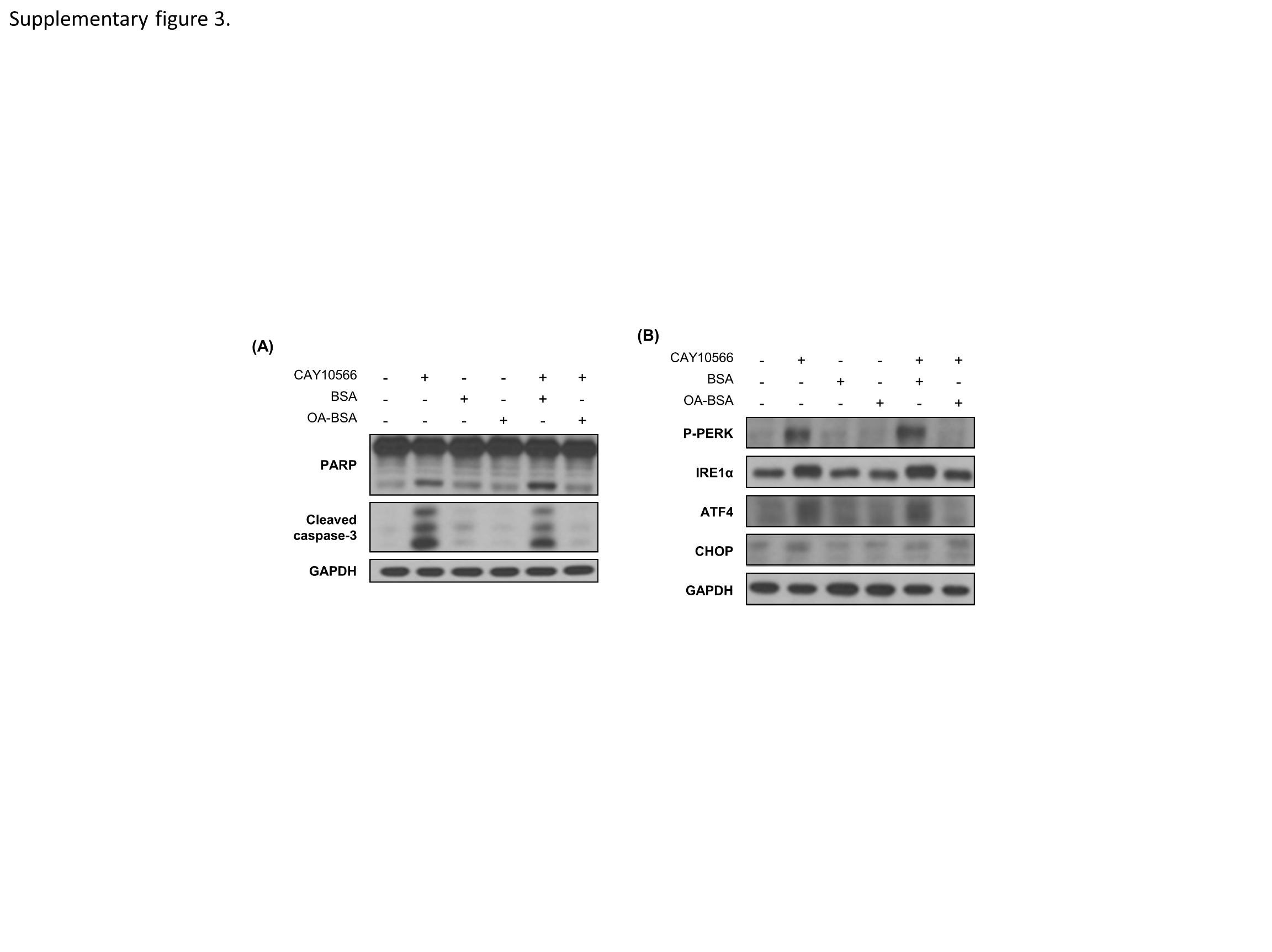


**Figure S2. Western blot analysis of exogenous oleic acid and CAY10566 treatment on apoptosis and ER stress markers.** (A) Assessment of apoptosis and (B) ER stress markers in PA-1 cells following treatment with exogenous oleic acid and CAY10566.
